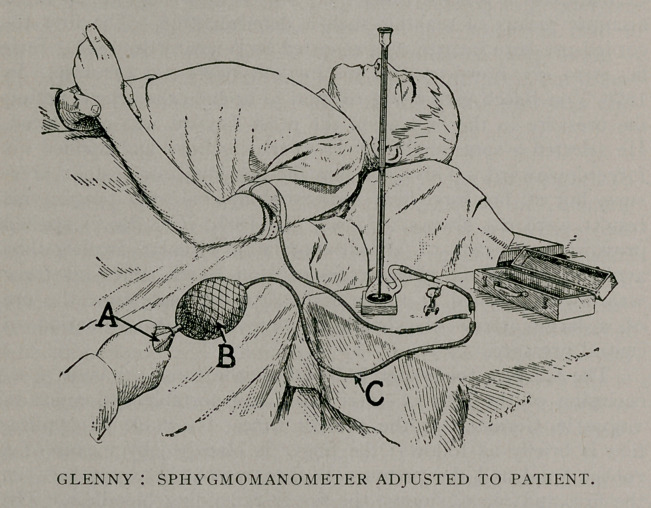# Accurate Determination of Blood Pressure as an Aid to Diagnosis1Read at the 68th annual meeting of the Medical Society of the County of Chemung, at Elmira, May 17, 1904.

**Published:** 1904-07

**Authors:** W. H. Glenny

**Affiliations:** Buffalo, N. Y., Assistant Attending Physician at the Buffalo General Hospital


### Accurate Determination of Blood Pressure as an Aid to Diagnosis.[1]

By W. H. GLENNY, M. D., Buffalo, N. Y.,
Assistant Attending Physician at the Buffalo General Hospital.

ESTIMATIONS of blood pressure were undoubtedly made by Hippocrates who probably used the fingers to obliterate the pulse. This is an excellent method and is of great value in the hands of a practised observer, but we now have much more accurate means of making such a determination. The first recorded pressure in man was reported by Vierordt in 1855. Suffice it to say, however, that his methods were not accurate. In 1876, Von Basch used a bag of fluid to occlude the artery, noting the pressure in the bag when the pulse beyond was obliterated. He selected a spot on the artery overlying bone and applied his force downward. This gave him the maximum or systolic pressure, but as his force was sometimes applied at a tangent his results were not always uniform. In 1896, Riva Rocci applied pressure by means of an air-containing circular armlet, thus avoiding one of the chief errors of Von Basch, since his force was always applied at right angle. At the same time the use of a large artery—the brachial—gave a better estimation of central pressure.

The Gärtner tonometer is based on the same principle, *i. e.*, occlusion of the artery. It is small and portable and much in vogue in Germany at the present time. Its mode of application is briefly as follows: the finger is blanched by means of a rubber band and the pressure is then removed, except between the first and second joints, the finger remaining bloodless. The pressure about the base of the finger is then lessened until flushing under the nail shows that the lumen of the artery is open. This records approximately the maximum pressure, but is open to objections. First, in order to make a second reading for verification the whole procedure must be repeated. Second, the arteries of the finger are subject to considerable vasomotor variation, and a high initial pressure to blanch the finger will give a lower reading than when an initial pressure of moderate degree is used.

My instrument is a slightly modified Riva Rocci sphygmomanometer made by Messrs. Eimer and Amend, of New York, who have followed suggestions of Cooke, of Baltimore, and made

---

1. Read at the 68th annual meeting of the Medical Society of the County of Chemung, at Elmira, May 17, 1904.

an article at once portable, easy of adjustment, and sufficiently accurate for clinical purposes. It is applied about the arm above the condyles and preferably below the biceps. Pressure is then applied by means of the bulb (*a*), until the radial pulse is obliterated, when the height of the column of mercury is noted. The mercury is then allowed to fall until the pulse is again palpable, and the mean between these two readings is taken as the estimate of the systolic blood pressure. The points at which the pulse is obliterated, and at which it is again felt, differ from each other only by a few m.m. and can be verified any number of times by

GLENNY: SPHYGMOMANOMETER ADJUSTED TO PATIENT.

squeezing or relaxing the reservoir bag (*b*). The whole process requires but a moment.

The diastolic or minimum pressure can be estimated in a different way. The walls of an artery make their greatest pulse wave excursion when the pressure of the tissues about the artery is equal to the pressure of the blood within, at a time when the arterial wall itself is under least tension. This time is of course at the end of diastoly, and this fact has been proven correct in numerous laboratory experiments by various observers. A device known as the Hill and Barnard sphygmometer makes use of this principle for estimating the diastolic pressure. An air-containing armlet, similar to the Riva Rocci, is connected by rigid tubing to a manometer which magnifies the pulse waves. As the pressure is increased within the armlet and tubes, the needle of the manometer begins to oscillate and the oscillations grow larger up to a certain point, when they again diminish in size and finally cease. The pressure at the time at which the greatest oscillation occurs is the diastolic blood pressure. A priori, one might suppose that the point at which oscillations cease would be the systolic pressure, but this is not the case, as the pulse beyond the constricted part of the artery is obliterated by a pressure considerably less than that at which oscillations of the manometer cease.

Stanton, of Philadelphia, has devised an instrument based on both of the above principles and with it measures both the systolic and diastolic pressure from which the mean pressure is easily estimated. His instrument is like the Riva Rocci, except that the armlet is wider and the tubing is composed of rigid instead of flexible rubber. This rigid tubing imparts oscillations to the column of mercury in the manometer and the pressure at which these are greatest is noted as the diastolic pressure. When the pulse in the artery beyond the point of compression is shut off the pressure is noted as the systolic pressure. The mean between these two readings being the mean blood pressure This instrument is made by the Arthur H. Thomas Company, of Philadelphia.

I find that my instrument, as shown in the cut, gives a reading of from 10 to 50 m.m. higher in a person with a fat arm than the same instrument with an armlet twice as wide. But the makers supply only the narrow armlet. This apparatus gives the maximum or systolic pressure, which for practical clinical purposes is all that is required. Howell and Brush have shown by a large number of experiments on animals in the pathological laboratory of the Johns Hopkins Hospital that the general trend of arterial tension is the same for either systolic, diastolic or mean pressure, except in hemorrhage, where there is probably more variation in the systolic than in the diastolic and hence than in the mean pressures.

In man with any of the above described instruments blood pressure varies with age and with the condition of the arteries. In general it may be said that in children blood pressure is much more uniform than in adults. Up to the age of two, a pressure of from 70 to 90 m.m. of mercury may be considered normal, and in children, 110. In adults where the arteries are soft and no diseased condition exists a pressure of 130 to 140 is about the average. Women have a pressure of perhaps 10 m.m. less than men, and a person resting in bed has about 10 m.m. less than when sitting or standing. Excitement or any stimulation, the inhalation of tobacco smoke or pinching the skin may raise arterial tension as much as 40 m.m. Therefore, in making an estimation outside influences must always be considered and avoided when possible. Arteriosclerosis plays an important rôle in blood tension estimation; the greater the sclerosis, the higher the tension estimation, probably because of the inelasticity of the artery walls. These general facts must be borne in mind when using such an instrument for purposes of diagnosis.

I want to call your attention to three diseases or conditions in which blood pressure observations, taken by means of one of the above described instruments are of value. I refer to uremia, intracranial hemorrhage and typhoid fever with its complications. No one now thinks that it is sufficient to note that a patient has a fever, but the degree of temperature is always taken with a thermometer and stated in terms of a known standard. Why, then, should we be satisfied with noting that the pulse tension is high when an accurate and definite statement can be made regarding it? In uremia, pulse tension is always high and in a doubtful case seen for the first time in a comatose condition the tension of the pulse is recognised as an important factor. The course of the disease can usually be foretold with considerable accuracy and when the tension falls in a case of uremia which is still showing such grave symptoms as headache, nausea or convulsions a favorable prognosis may be made, although all other clinical symptoms remain unchanged. And this lowering of pulse tension may be quite imperceptible to the unaided finger of even the most skilled observer. In like manner therapeutic measures may be commenced before such active signals as convulsions have manifested themselves and the severity of the attack undoubtedly shortened by beginning treatment earlier than otherwise would be possible. These facts apply as well in puerperal eclampsia as in uremia.

*Intracranial hemorrhage.*—The brain is the only organ of the body which has not its own vasomotor mechanism. It is believed that when the brain needs more blood the vessels of the body are constricted through the central nervous system, thus sending more blood to the brain as less is allowed to pass through the general systemic circulation. After an intracranial hemorrhage the brain becomes anemic from pressure of the clot and the vasomotor centers contract the arteries of the body in an endeavor to send more blood to the brain. The pulse tension becomes high and again, as in uremia, an accurate estimation of this is of value. In this case in deciding whether or not trephining is necessary. Another point to be taken into consideration as an indication of intracranial hemorrhage is a pressure which shows from moment to moment great alterations in level. A case in point being one operated upon by Dr. Finney, of Baltimore.

A young man received a blow on the head and, as I recall the case, there was no indication other than the history; the fact that the patient on the second day complained of more headache than on the first, and that the blood pressure showed great variation, changing in a moment over level of 30 or 40 m.m., but at no time remaining uniformly high. Dr. Finney operated and removed a small extradural clot, the patient making a good recovery.

I feel some hesitancy about mentioning this case, as I report it entirely from memory and the operation took place two years ago, but I wish to bring out the importance of oscillation in blood pressure. Dr. Harvey Cushing, of Baltimore, who was one of the pioneers in the use of blood pressure estimations, in speaking of their value in cranial trauma says: "In conjunction with other symptoms a progressive increase in arterial pressure or a high degree of the same which has been already reached or a pressure which exhibits from moment to moment great alterations in level may be taken as a certain indication of the advisability of early operative intervention."

*Typhoid fever.*—The blood pressure in typhoid fever is uniformly low except in complications. In 115 cases which Geo. W. Crile, of Cleveland, reports, the highest pressure noted of any uncomplicated case was 138 m.m.,—the lowest, 74 m.m. The average pressure for first week, 115 m.m.; second week, 106 m.m.; third week, 102 m.m.; fourth week, 96 m.m.; fifth week, 98 m.m. This low tension is evident in the dicrotic pulse, so commonly seen in typhoid. If in a patient suspected of having typhoid fever, with soft arteries and no kidney complications, we find a blood pressure of 140 m.m. of mercury, it is very strong evidence that the patient is not suffering from that disease—and such an accurate estimate cannot be made with the unaided finger. The pressure is remarkably uniform in typhoid and a sudden fall of 20 to 30 m.m. in a patient under constant observation may be taken as a strong indication of hemorrhage and steps taken accordingly. Its value here, however, is not as great as in perforation where a correct diagnosis and a prompt operation often means life to a patient who would otherwise have a surely fatal termination of his illness. In peritonitis the blood pressure is always high except just before death, or after great shock. In a series of twenty cases reported by Dr. Crile, made with the same instrument and by the same observer as the typhoid series just mentioned, the highest pressure was 208, the lowest 156 m.m.,—the average of all the cases being 166 m.m. Whereas the average of the typhoid cases was 104 m.m. Among these twenty cases, there were five typhoid perforations, the blood pressure before and after perforation being as follows:

| | | | | | |
|---|---|---|---|---|---|
| Before | 84 | 116 | 116 | — | — |
| After | 110 | 180 | 165 | 165 | 208 |

All of these figures are reported by Dr. Crile in the *Journal of the American Medical Association,* May 9, 1903. For more than a year I have been estimating with the Riva Rocci instrument the blood pressure of nearly all typhoid patients in the wards of the Buffalo General Hospital and can corroborate Dr. Crile's figures. The highest pressure of uncomplicated cases with soft arteries, coming under my notice, has been 132 m.m.

Dr. J. B. Briggs reported in the *Boston Medical and Surgical Journal,* September 24, 1903, two cases from the wards of the Johns Hopkins Hospital which are such striking illustrations of the value of blood pressure estimations in typhoid perforation that I will give them in some detail, as the second one came under my own observation:

Case I.—A young man entered the hospital in the third week of an attack of typhoid. On entrance he was very toxic though not in stupor. Blood pressure for ten days after entrance ranged from 98 to 110. At eight o'clock on the night in which perforation occurred, his blood pressure was 104; at midnight it was 144. At that time he complained of no pain and his abdomen was everywhere soft and natural, deep pressure causing no distress. His leukocyte count below 6,000. At 4 a. m., when being taken from a tub, he gave a cry of pain, the pain being located below the umbilicus. When seen at that time his knees were drawn up and there was extreme tenderness in the right iliac fossa, rigid lower right rectus, and a suggestion of muscle spasm. Blood pressure 150, leukocytes still below 6,000.

He was operated upon at 8 a. m. There were two perforations with rapidly spreading peritonitis which the surgeons estimated to have been present eight or ten hours, certainly more than four hours, which was the time which had elapsed since the first sudden starting of pain. It was suggested that in this case perforation had occurred before twelve o'clock, the time at which the high arterial tension was first noted, but that it had been confined within coils of the intestine touching only the visceral peritoneum and that not until four o'clock, the time of the pain, did the peritonitis spread to the parietal walls.

Case II.—That of a woman who had been in the hospital fifty-seven days. She had had two relapses and five hemorrhages, but no complications for three weeks prior to the date of operation. She had marked toxemia, slight delirium, and slight abdominal pain, never well localised, for several weeks. The leukocyte counts had remained constantly low, the abdomen was somewhat full; but there was no tympanites until three days before operation, when this developed with slightly increased tenderness of the abdomen, never localised. Liver dulness was obliterated the day before operation and there was considerable increase of pain, though never very severe; not enough so that the thighs were flexed. Her facial expression was quite drawn. On the day of operation the leukocytes rose rapidly to 17,000, never before in many counts having been above 8,000. There was no muscle spasm, but tenderness was increased and respiratory movements more limited so that an operation was decided upon, in spite of the fact that blood pressure remained constantly between 115 and 122 and that peripheral stimulation of the skin and central stimulation with strychnia caused a rise in pressure, showing that the vasomotor mechanism was active. Operation revealed no peritonitis or perforation, although over the base of two ulcers there was a slight amount of fibrin. In this case hemorrhage was considered but excluded because the temperature and blood pressure had not fallen.

I do not wish to intimate from the history of these cases that estimation of the blood pressure is the one and only thing to be taken into consideration in making a diagnosis of typhoid perforation, but I consider that it should take its place as an important factor in forming an opinion and believe that conclusions drawn from it are of more import than from leukocytosis or distension, and that it should be ranked with muscle spasm, muscle rigidity, general expression and sudden pain.

185 Summer Street.

## Figures and Tables

**Figure f1:**